# Prognostic nutritional index as a predictor of anastomotic leakage after rectal cancer surgery

**DOI:** 10.3389/fsurg.2026.1825596

**Published:** 2026-05-15

**Authors:** Kazım Duman

**Affiliations:** Ministry of Health, Sancaktepe Şehit Dr. İlhan Varank Training and Research Hospital, İstanbul, Türkiye

**Keywords:** anastomotic leakage, neoadjuvant therapy, prognostic nutritional index, rectal cancer, risk stratification

## Abstract

**Background and objectives:**

Anastomotic leakage remains a major complication following rectal cancer surgery. Neoadjuvant therapy, while improving oncological outcomes, may negatively impact nutritional and immunological status. This study aimed to evaluate the predictive value of the Prognostic Nutritional Index (PNI) for anastomotic leakage in locally advanced rectal cancer patients treated with neoadjuvant therapy.

**Materials and methods:**

This retrospective study included 123 patients with locally advanced rectal cancer who underwent neoadjuvant therapy followed by curative resection with primary anastomosis between 2018 and 2024. PNI was calculated using preoperative serum albumin and lymphocyte count. ROC curve analysis determined the optimal PNI cutoff value. Univariate and multivariate logistic regression analyses identified independent predictors of anastomotic leakage.

**Results:**

Anastomotic leakage occurred in 19 patients (15.4%). The optimal PNI cutoff was 51.25 (AUC = 0.756, sensitivity 78.9%, specificity 67.3%). In multivariate analysis, PNI was an independent predictor of anastomotic leakage (OR = 0.809, 95% CI: 0.696–0.941, *p* = 0.006). Coronary artery disease (*p* = 0.038) and intraoperative blood transfusion (*p* = 0.001) were also identified as significant independent risk factors for anastomotic leakage.

**Conclusions:**

Preoperative PNI is an independent predictor of anastomotic leakage in rectal cancer patients following neoadjuvant therapy. PNI may serve as a practical tool for preoperative risk stratification and guide clinical decision-making regarding nutritional optimization and surgical planning.

## 1 Introduction

Colorectal cancer remains a significant global health problem, being the third most commonly diagnosed cancer worldwide and the second leading cause of cancer-related deaths (1). In approximately one-third of these patients, the tumor is localized in the rectum (2). Anastomotic leakage (AL) in rectal surgery is one of the most critical complications, occurring in 2.8%–30% of patients (3). Despite advances in surgical techniques and perioperative care, AL continues to represent a major clinical challenge. Beyond its immediate impact on short-term morbidity—including increased reoperation rates, prolonged hospital stays, delayed initiation of adjuvant treatment, and prolonged stoma-related conditions—AL has also been shown to adversely affect long-term oncological outcomes, including local recurrence rates and cancer-specific survival (4, 5).

The management of locally advanced rectal cancer has undergone substantial evolution over the past decades. Neoadjuvant chemoradiotherapy (nCRT) has been established as the standard of care, demonstrating benefits in terms of tumor downstaging, sphincter preservation, and local recurrence reduction (6). More recently, total neoadjuvant therapy (TNT) has gained increasing acceptance. TNT has shown advantages including improved pathological complete response rates, better compliance with systemic therapy, and potentially improved oncological outcomes (7, 8). However, the intensification of neoadjuvant treatment raises concerns regarding its impact on surgical complications.

The effect of neoadjuvant therapy, particularly TNT, on the development of AL remains a subject of ongoing debate. While some studies suggest that prolonged neoadjuvant treatment may increase tissue fibrosis and impair anastomotic healing, others have reported no significant difference in AL rates between patients receiving conventional nCRT and those undergoing TNT (9, 10). This heterogeneity in the literature highlights the need for further investigation into the factors contributing to AL in this specific patient population.

Several risk factors have been identified as contributing to the development of AL following rectal surgery. These factors are commonly categorized as patient-related, tumor-related, and surgery-related variables. Patient-related factors include male sex, advanced age, obesity, malnutrition, diabetes mellitus, smoking, and immunosuppressive conditions. Tumor-related factors encompass low tumor location, advanced tumor stage, and tumor size. Surgery-related factors include prolonged operative time, intraoperative blood loss, contamination, and the level of the anastomosis from the anal verge (11). In addition to these conventional risk factors, various nutritional and inflammatory indices—such as the Prognostic Nutritional Index (PNI), Neutrophil-to-Lymphocyte Ratio (NLR), and Platelet-to-Lymphocyte Ratio (PLR)—have been investigated as potential predictors of AL, reflecting the systemic inflammatory and nutritional status of patients (12, 13).

Among the factors influencing postoperative outcomes, the Prognostic Nutritional Index (PNI), calculated using serum albumin levels and peripheral blood lymphocyte count, has emerged as a reliable and easily accessible marker reflecting both nutritional and immunological status. Originally developed by Onodera et al. for predicting surgical risk in gastrointestinal cancer patients, PNI has since been validated as a prognostic indicator in various malignancies, including gastric and colorectal cancer (8, 9). Low PNI values have been associated with increased postoperative complications, poor oncological outcomes, and impaired wound healing (12, 14, 15).

However, two important limitations characterize the existing literature. First, studies evaluating PNI specifically in rectal cancer—as distinct from colorectal cancer broadly—remain limited, and the generalizability of findings from mixed cohorts to this specific anatomical and oncological context is uncertain. Second, and more critically, prior investigations have largely included heterogeneous surgical populations that combine patients with and without neoadjuvant therapy, without accounting for the distinct nutritional and immunological alterations induced by neoadjuvant treatment. Neoadjuvant chemoradiotherapy, and particularly TNT, may substantially impair a patient's systemic nutritional and immune reserve, independently modulating the risk of anastomotic complications. To our knowledge, no study has specifically examined the predictive value of preoperative PNI for anastomotic leakage in a homogeneous cohort of locally advanced rectal cancer patients treated with neoadjuvant therapy—a clinically relevant knowledge gap given the increasing adoption of intensified neoadjuvant protocols.

In this study, we aimed to evaluate the predictive value of preoperative PNI for anastomotic leakage in patients with locally advanced rectal cancer who received neoadjuvant therapy, and to identify independent preoperative risk factors for this complication (16).

## 2 Materials and methods

### 2.1 Study design and ethical approval

This retrospective cohort study evaluated patients who completed neoadjuvant therapy for locally advanced rectal cancer and subsequently underwent low anterior resection with defunctioning loop ileostomy at the Department of General Surgery, Sancaktepe Şehit Dr. İlhan Varank Training and Research Hospital between January 2018 and December 2024. The study was approved by the local Ethics Committee (date: 11.02.2026; approval no.: 2026/87). As the study involved retrospective analysis of existing medical records, the requirement for individual informed consent was waived. The study was conducted in accordance with the Declaration of Helsinki and applicable ethical standards, and patient privacy and confidentiality were maintained throughout.

The primary outcome was identification of independent risk factors for clinically significant AL [International Study Group of Rectal Cancer (ISREC) grade B and C] (16).

### 2.2 Inclusion criteria

To enable a homogeneous evaluation of predictive factors for anastomotic leakage in rectal cancer patients undergoing low anterior resection after neoadjuvant therapy, specific inclusion criteria were defined. These were: age between 18 and 80 years; histopathologically confirmed diagnosis of rectal adenocarcinoma; tumor located within 15 cm from the anal verge; clinical stage cT3–T4 and/or cN+ disease; receipt of long-course radiotherapy (50.4 Gy in 28 fractions) as part of either conventional neoadjuvant chemoradiotherapy or total neoadjuvant therapy (TNT) protocols; completion of the planned neoadjuvant treatment; performance of total mesorectal excision (TME); and creation of a defunctioning loop ileostomy at the time of resection (6, 7).

### 2.3 Exclusion criteria

Exclusion criteria were defined to allow specific assessment of prognostic factors and to minimize heterogeneity. Patients were excluded if they: had a diagnosis other than rectal adenocarcinoma on final histopathological examination; were younger than 18 years or older than 80 years; had positive proximal, distal, or circumferential resection margins; underwent emergency surgery due to perforation, bleeding, or acute mechanical obstruction; had neoadjuvant therapy discontinued due to treatment-related complications or emergency conditions; completed neoadjuvant therapy after creation of a diverting stoma under emergency conditions; had a history of short-course radiotherapy; did not undergo defunctioning loop ileostomy at the time of resection; had a prior history of pelvic radiotherapy for other indications; underwent additional organ resections due to synchronous lesions or locally advanced disease requiring multivisceral resection; had a positive intraoperative air-leak test; required reinforcing sutures at the anastomotic site; or had anastomosis performed with a 25-mm circular stapler.

### 2.4 Data collection

The study was initiated by screening the records of patients who underwent low anterior resection at the center between January 2018 and December 2024. Demographic data, including age, sex, body mass index (BMI), comorbidities, and American Society of Anesthesiologists (ASA) physical status classification were recorded. Preoperative laboratory parameters included hemoglobin, albumin, neutrophil count, lymphocyte count, and platelet count. From these values, the neutrophil-to-lymphocyte ratio (NLR), platelet-to-lymphocyte ratio (PLR), and prognostic nutritional index (PNI) were calculated. PNI was calculated using the formula: 10 × serum albumin (g/dL) + 0.005 × total lymphocyte count (/mm^3^) (12). Laboratory reference ranges were defined as follows: hemoglobin 12.0–17.5 g/dL, serum albumin 3.5–5.0 g/dL, lymphocyte count 1,000–4,800/mm^3^, neutrophil count 1,800–7,500/mm^3^, and platelet count 150,000–400,000/mm^3^ (17–19).

Treatment-related variables included neoadjuvant therapy protocol (conventional chemoradiotherapy vs. total neoadjuvant therapy), interval from completion of radiotherapy to surgery, operative time, intraoperative blood loss, need for intraoperative blood transfusion, surgical approach (open vs. laparoscopic), level of inferior mesenteric artery ligation (high vs. low), and distance of the anastomosis from the anal verge.

Pathological data included tumor location within the rectum, pathological tumor and nodal stages (ypT and ypN), and tumor regression grade (TRG) assessed according to the Ryan classification system. Length of hospital stay and 30-day postoperative outcomes were also recorded (20).

### 2.5 Preoperative management

Following histopathological confirmation of rectal adenocarcinoma, clinical staging was performed in all patients using pelvic magnetic resonance imaging (MRI) and thoracoabdominal computed tomography (CT). Patients with tumor extension beyond the muscularis propria into the mesorectal fat (cT3–cT4) and/or clinically positive lymph nodes (cN+), as well as those with MRI findings of threatened or involved circumferential resection margin (CRM ≤1 mm) and/or internal sphincter involvement, were considered to have locally advanced disease and referred for neoadjuvant treatment (21).

Patients received either conventional long-course chemoradiotherapy (CRT) consisting of 50.4 Gy in 28 fractions with concurrent fluoropyrimidine-based chemotherapy (intravenous 5-fluorouracil or oral capecitabine), or TNT according to the PRODIGE-23 protocol. Surgery was scheduled at least 6 weeks after completion of radiotherapy to allow for tumor response assessment and patient recovery.

### 2.6 Surgical management

Preoperative laboratory tests were performed in all patients one week before surgery. Parameters potentially associated with anastomotic leakage risk (e.g., hemoglobin, albumin) and markers indicating acute inflammatory or infectious conditions were evaluated. In patients with evidence of an acute inflammatory or infectious process, surgery was postponed until clinical resolution.

Preoperative packed red blood cell transfusion was administered when hemoglobin levels were <10 g/dL in patients with comorbidities or <7 g/dL in patients without comorbidities. The neutrophil-to-lymphocyte ratio (NLR), platelet-to-lymphocyte ratio (PLR), and prognostic nutritional index (PNI) were calculated from the preoperative laboratory results obtained within one week before surgery.

All operations were performed at a single center by board-certified surgeons with at least 10 years of experience in colorectal surgery. Total mesorectal excision (TME) was performed using either an open or laparoscopic approach. A defunctioning loop ileostomy was created in all patients. All anastomoses were constructed as side-to-end colorectal anastomoses using a 29-mm circular stapler. An intraoperative air-leak test was routinely performed by transanal air insufflation with the anastomosis submerged under saline solution. Patients with a positive air-leak test or those requiring reinforcing sutures at the anastomotic site were excluded from the study to ensure technical standardization. High ligation of the inferior mesenteric artery was performed in all patients.

Postoperatively, early oral nutrition was initiated unless clinically contraindicated. Patients were followed for 30 days postoperatively for the development of anastomotic leakage and other complications. Postoperative follow-up was conducted through daily clinical assessment during the inpatient period. Routine laboratory tests, including complete blood count and biochemical panels, were performed twice weekly. The development of anastomotic leakage was suspected in the presence of unexplained and persistent fever or abdominal pain not attributable to other causes, sustained elevation of inflammatory markers, or purulent discharge from drains or wound sites. In cases of suspected AL, computed tomography with rectal and intravenous contrast was performed. When clinical signs were overt, no additional diagnostic workup was deemed necessary; in cases of ongoing suspicion without definitive radiological findings, rectoscopic examination was performed. For patients discharged prior to 30 days, AL-related events were identified through systematic review of hospital readmission records, discharge summaries, imaging reports, and colonoscopic examination records.

### 2.7 Definition and grading of anastomotic leakage

Anastomotic leakage was defined according to the International Study Group of Rectal Cancer (ISREC) criteria as a defect of the intestinal wall integrity at the anastomotic site leading to communication between the intra- and extraluminal compartments. A pelvic abscess adjacent to the anastomosis was also considered as anastomotic leakage. The diagnosis was established based on clinical presentation (fever, pelvic pain, purulent or fecal discharge from the drain, peritonitis), elevated inflammatory markers (C-reactive protein, white blood cell count), and confirmed by digital rectal examination, rectoscopy, computed tomography with rectal contrast, or a combination of these modalities.

The severity of anastomotic leakage was graded according to the ISREC classification: Grade A, requiring no change in management; Grade B, requiring active therapeutic intervention (antibiotics, percutaneous or transanal drainage) but manageable without relaparotomy; and Grade C, requiring surgical reintervention. For the purposes of this study, clinically significant anastomotic leakage was defined as ISREC Grade B and C, which constituted the primary outcome. Overall anastomotic leakage (Grades A, B, and C combined) was analyzed as a secondary outcome.

### 2.8 Statistical analysis

Statistical analyses were performed using IBM SPSS Statistics version 25.0 (IBM Corp., Armonk, NY, USA). Normality was assessed using the Kolmogorov–Smirnov test. Continuous variables were compared using the independent samples *t*-test or Mann–Whitney *U*-test, as appropriate, and presented as mean ± standard deviation (sd) or median and interquartile range (IQR). Categorical variables were compared using the chi-square or Fisher's exact test and expressed as frequencies and percentages. ROC curve analysis was performed to determine optimal cut-off values for significant continuous variables associated with anastomosis leak, with calculation of AUC, sensitivity, and specificity. Significant variables in univariate analysis were included in multivariate binary logistic regression to identify independent predictors of AL. A *p*-value < 0.05 was considered statistically significant.

## 3 Results

**Figure 1:**
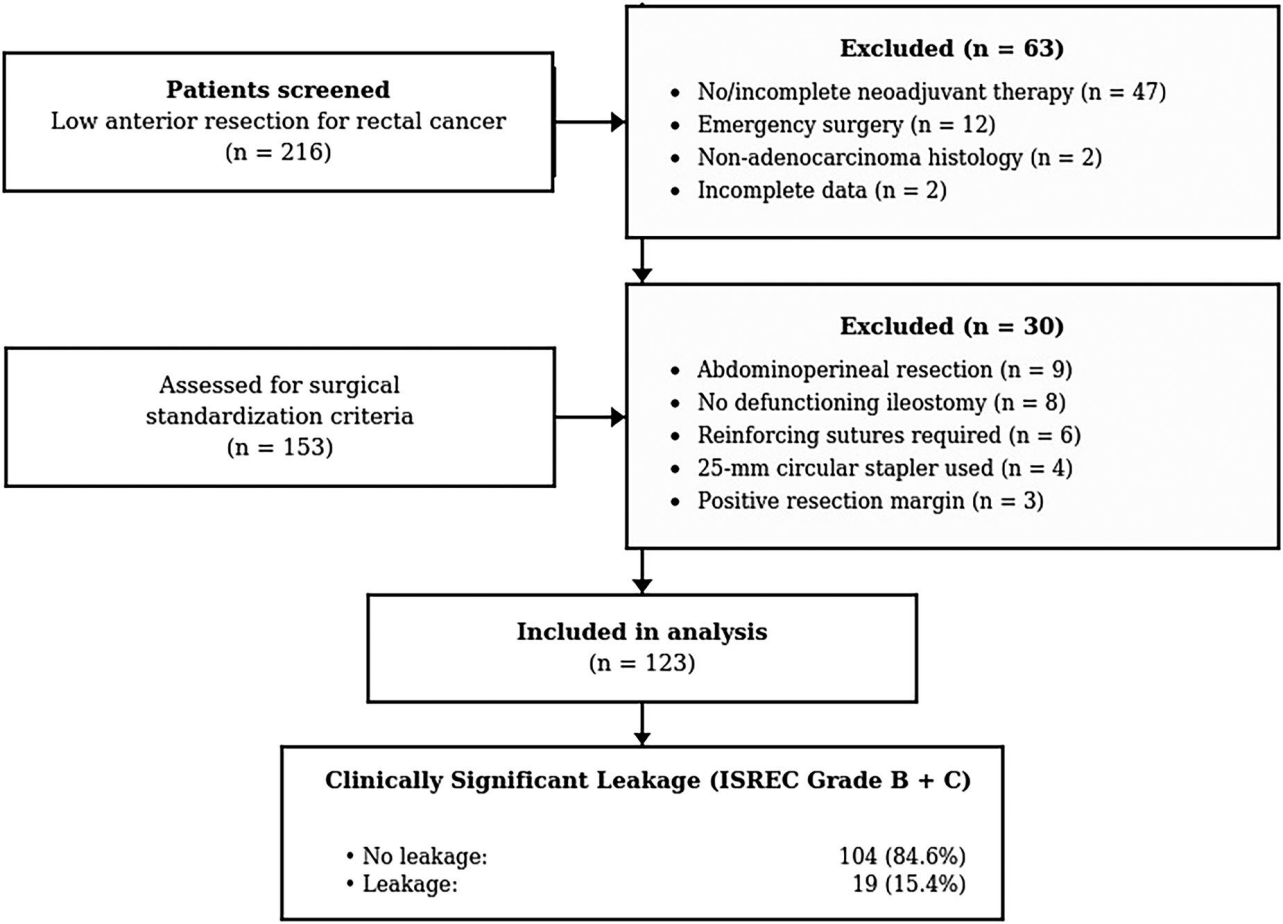
Flowchart of patient enrollment.

Between January 2018 and December 2024, a total of 216 patients who underwent low anterior resection for rectal cancer at our institution were screened for eligibility. In the first step, 63 patients were excluded: no neoadjuvant therapy or failure to complete the planned treatment (*n* = 47), emergency surgery (*n* = 12), non-adenocarcinoma histology (*n* = 2), and incomplete data (*n* = 2). The remaining 153 patients were further evaluated for surgical standardization criteria. An additional 30 patients were excluded: abdominoperineal resection (*n* = 9), no defunctioning loop ileostomy (*n* = 8), reinforcing sutures required at the anastomotic site (*n* = 6), use of 25-mm circular stapler (*n* = 4), and positive resection margin (*n* = 3). Finally, 123 patients were included in the analysis. AL occurred in 19 patients (15.4%) (Figure 1).

When the groups were compared according to the development of AL, there were 19 patients with AL (AL+) and 104 patients without AL (AL−). In the demographic assessment, ASA I (10.6% vs. 5.3%) and ASA II (46.2% vs. 15.8%) classifications were more frequent in the AL− group, whereas ASA III (43.3% vs. 63.2%) and ASA IV (0.0% vs. 15.8%) were more common in the AL+ group (*p* < 0.001). In addition, the presence of coronary artery disease (CAD) was significantly higher in the AL+ group (36.8% vs. 16.3%, *p* = 0.038).

Regarding treatment strategies, the rate of anastomotic leakage was significantly higher in patients who received total neoadjuvant therapy (TNT) than in those who received chemoradiotherapy (CRT) (63.2% vs. 26.0%, *p* = 0.001). Conventional surgery was performed more frequently in the AL+ group (57.9% vs. 32.7%, *p* = 0.036). The need for intraoperative transfusion was higher in the AL+ group (31.6% vs. 6.7%, *p* = 0.001). Preoperative hemoglobin [12 (11.2–13.1) vs. 13 (12.8–13.5) g/dL, *p* = 0.034], albumin [3.79 (3.50–4.00) vs. 4.25 (4.18–4.36) g/dL, *p* < 0.001], and PNI values [48.90 (47.75–52.35) vs. 53.97 (52.60–55.95), *p* < 0.001] were lower in the AL+ group. Operative time was longer in the AL+ group [220 (210–280) vs. 210 (210–230) min, *p* = 0.045), and the anastomotic distance from the anal verge was shorter [5 (5–6) vs. 6 (6–7) cm, *p* = 0.017]. Length of hospital stay was significantly longer in the AL+ group [15 (12–23) vs. 8 (8–9) days, *p* < 0.001]. No significant differences were observed between the groups in terms of other demographic, pathological, or surgery-related parameters (Table 1).

**Table 1:** Comparison of demographic, clinical, and pathological characteristics between patients with and without anastomotic leakage.

Variables	Parameters	AL—(*n* = 104)	AL + (*n* = 19)	*p* ^a^
		(*n*, %)	(*n*, %)	
Gender	Male	63 (60.6%)	10 (52.6%)	0.517
	Female	41 (39.4%)	9 (47.4%)	
ASA Score	ASA1	11 (10.6%)	1 (5.3%)	**<0** **.** **001**
	ASA2	48 (46.2%)	3 (15.8%)	
	ASA3	45 (43.3%)	12 (63.2%)	
	ASA4	0 (0%)	3 (15.8%)	
HT	Yes	43 (41.3%)	10 (52.6%)	0.361
DM	Yes	26 (25.0%)	4 (21.1%)	0.713
COPD	Yes	8 (7.7%)	3 (15.8%)	0.255
CAD	Yes	17 (16.3%)	7 (36.8%)	**0** **.** **038**
Smoking	Yes	33 (31.7%)	9 (47.4%)	0.186
Neoadjuvant	CRT	77 (74.0%)	7 (36.8%)	**0** **.** **001**
	TNT	27 (26.0%)	12 (63.2%)	
Surgery	Laparoscopic	70 (67.3%)	8 (42.1%)	**0** **.** **036**
	Conventional	34 (32.7%)	11 (57.9%)	
Transfusion	Yes	7 (6.7%)	6 (31.6%)	**0** **.** **001**
cT Stage	T_1_	1 (1.0%)	0 (0%)	0.808
	T_2_	9 (8.7%)	1 (5.3%)	
	T_3_	75 (72.1%)	13 (68.4%)	
	T_4_	19 (18.3%)	5 (26.3%)	
cN Stage	N_0_	62 (59.6%)	8 (42.1%)	0.475
	N_1_	28 (26.9%)	8 (42.1%)	
	N_2_	7 (6.7%)	2 (10.5%)	
	N_3_	7 (6.7%)	1 (5.3%)	
cTNM Stage	Stage 2	62 (59.6%)	8 (42.1%)	0.156
	Stage 3	42 (40.4%)	11 (57.9%)	
yP T Stage	T_0_	9 (8.7%)	0 (0%)	0.576
	T_1_	27 (26.0%)	4 (21.1%)	
	T_2_	28 (26.9%)	7 (36.8%)	
	T_3_	25 (24.0%)	6 (31.6%)	
	T_4_	15 (14.4%)	2 (10.5%)	
yP N Stage	N_0_	63 (60.6%)	7 (36.8%)	0.707
	N_1_	31 (29.8%)	5 (26.3%)	
	N_2_	8 (7.7%)	1 (5.3%)	
	N_3_	8 (7.7%)	0 (0%)	
ypTNM Stage	Stage 0	7 (6.7%)	0 (0%)	0.456
	Stage1	35 (33.7%)	9 (47.4%)	
	Stage2	25 (24.0%)	3 (15.8%)	
	Stage 3	37 (35.6%)	7 (36.8%)	
TRG Ryan	0	8 (7.7%)	0 (0%)	0.638
	1	30 (28.8%)	6 (31.6%)	
	2	39 (37.5%)	7 (36.8%)	
	3	27 (26.0%)	6 (31.6%)	
		Mean ± sd	Mean ± sd	p^b^
BMI	Kg/m^2^	27.32 ± 0.37	26.63 ± 0.52	0.056
		Median (IQR)	Median (IQR)	p^c^
Age	years	63 (62–65)	68 (58–72)	0.273
Hb	g/dL	13 (12.8–13.5)	12 (11.2–13.1)	**0** **.** **034**
Alb	g/dL	4.25 (4.18–4.36)	3.79 (3.50–4.00)	**<0** **.** **001**
NLR	Ratio	2.48 (2.20–2.73)	2.41 (2.25–2.87)	0.972
PNI	Index	53.97 (52.60–55.95)	48.90 (47.75–52.35)	**<0** **.** **001**
Operation Time	Minutes	210 (210–230)	220 (210–280)	**0** **.** **045**
DFAV	Cm	6 (6–7)	5 (5–6)	**0** **.** **017**
RTI	Days	26 (25–29)	57 (55–59)	0.180
LOS	Days	8 (8–9)	15 (12–23)	**<0** **.** **001**

AL, anastomotic leakage; ASA, American Society of Anesthesiologists; HT, hypertension; DM, diabetes mellitus; COPD, chronic obstructive pulmonar*y* disease; CAD, coronary artery disease; CRT, chemoradiotherapy; TNT, total neoadjuvant therapy; BMI, body mass index; Hb, hemoglobin; Alb, albumin; NLR, neutrophil-to-lymphocyte ratio; PNI, Prognostic Nutritional Index; DFAV, distance from anal verge; RTI, radiotherapy-to-surgery interval; LOS, length of hospital stay; TRG, tumor regression grade; IQR, interquartile range; SD, standard deviation.

a Chi-Square Test.

b Independent Samples *T-*Test.

c Mann–Whitney *U*-Test.

Bold *p* values indicate statistical significance (*p* < 0.05).

**Figure 2:**
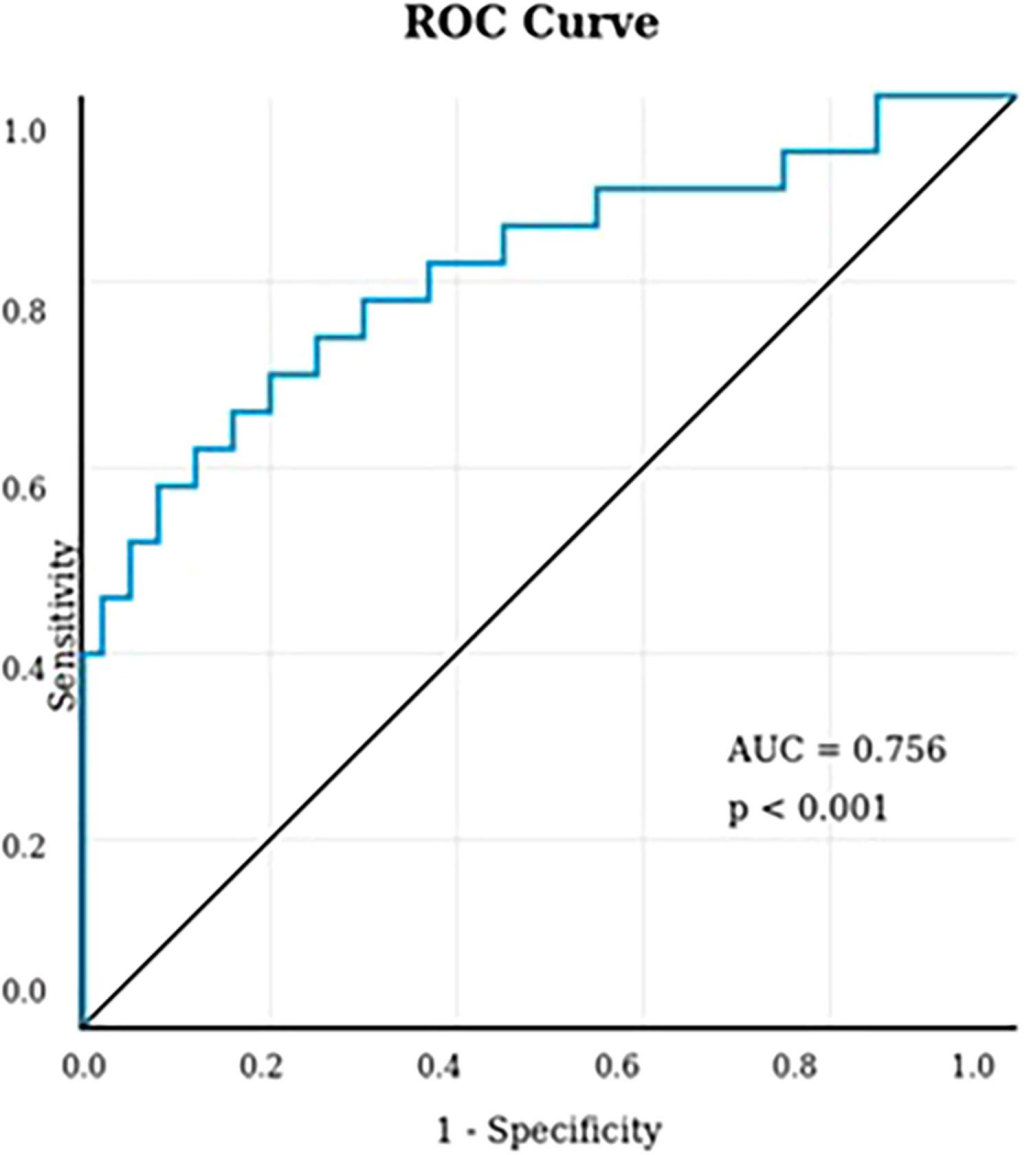
ROC analysis of PNI in AL.

Because PNI was significantly associated with both overall AL+, ROC analysis was performed to determine the optimal cutoff value. PNI was identified as a significant marker (AUC = 0.756 ± 0.049, 95% CI: 0.661–0.852, *p* < 0.001). Using the Youden index, the optimal cutoff value was 51.25, yielding a sensitivity of 71.2% and a specificity of 57.9% (Figure 2) (Table 2).

**Table 2:** ROC curve analysis of prognostic nutritional Index for predicting overall and clinically significant anastomotic leakage.

	AL +						
Variables	AUC	SA_AUC_	95% CI	Cutoff	Sensitivity%	Specificity%	*p*
PNI	0.756	0.049	0.661–0.852	51.25	%78.9	%67.3	**<0** **.** **001**

ROC, receiver operating characteristic; AUC, area under the curve; SAAUC, standard area under the curve; CI, confidence interval; AL, anastomotic leakage; PNI, Prognostic Nutritional Index. Bold *p* values indicate statistical significance (*p* < 0.05).

When the TNT (*n* = 39) and conventional CRT (*n* = 84) groups were compared, ASA III (53.8% vs. 7.7%) and ASA IV (7.7% vs. 0.0%) classifications were more frequent in the TNT group. In clinical nodal (cN) staging, the proportion of N0 disease was higher in the TNT group (69.2% vs. 51.2; *p* = 0.024). Regarding pathological T staging, the TNT group had a higher pCR (ypT0) rate (12.8% vs. 4.8%) and a lower ypT4 rate (2.6% vs. 19.0%, *p* = 0.039).

In pathological TNM staging, the proportion of Stage 0–I disease was markedly higher in the TNT group (61.5% vs. 32.2%), whereas Stage III disease was more frequent in the CRT group (42.9% vs. 20.5, *p* = 0.007). As expected, the interval between radiotherapy and surgery was longer in the TNT group [63 (60–66) vs. 24 (24–25) days, *p* < 0.001]. Operative time was also longer in the TNT group [220 (210–260) vs. 212.5 (205–220) min, *p* = 0.041], and length of hospital stay was longer [11 (9–14) vs. 8 (8–9) days, *p* = 0.007]. No significant differences were observed between the groups with respect to other demographic, clinical, or pathological parameters (Table 3).

**Table 3:** Comparison of demographic, clinical, and pathological characteristics between total neoadjuvant therapy and conventional chemoradiotherapy groups.

Variables	Parameters	TNT (*n* = 39)	CRT (*n* = 84)	*P* ^a^
		(*n*,%)	(*n*,%)	
Gender	Male	21 (53.8%)	52 (61.9%)	0.397
	Female	18 (46.2%)	32 (38.1%)	
ASA Score	ASA 1	3 (7.7%)	9 (10.7%)	**0** **.** **030**
	ASA 2	12 (30.8%)	39 (46.4%)	
	ASA 3	21 (53.8%)	36 (42.9%)	
	ASA 4	3 (7.7%)	0 (0%)	
Smoking	Yes	15 (38.5%)	27 (32.1%)	0.492
HT	Yes	18 (46.2%)	35 (41.7%)	0.640
DM	Yes	9 (23.1%)	21 (25.0%)	0.817
COPD	Yes	2 (5.1%)	9 (10.7%)	0.312
CAD	Yes	7 (17.9%)	17 (20.2%)	0.766
Surgery	Laparoscopic	25 (64.1%)	53 (63.1%)	0.914
	Open	14 (35.9%)	31 (36.9%)	
Transfusion	Yes	5 (12.8%)	8 (9.5%)	0.580
cT Stage	T1	1 (2.6%)	0 (0%)	0.201
	T2	1 (2.6%)	9 (10.7%)	
	T3	30 (76.9%)	58 (69.0%)	
	T4	7 (17.9%)	17 (20.2%)	
cN Stage	N0	27 (69.2%)	43 (51.2%)	**0** **.** **024**
	N1	12 (30.8%)	24 (28.6%)	
	N2	0 (0%)	9 (10.7%)	
	N3	0 (0%)	8 (9.5%)	
cTNM Stage	Stage II	27 (69.2%)	43 (51.2%)	0.060
	Stage III	12 (30.8%)	41 (48.8%)	
ypT Stage	T0	5 (12.8%)	4 (4.8%)	**0** **.** **039**
	T1	8 (20.5%)	23 (27.4%)	
	T2	15 (38.5%)	20 (23.8%)	
	T3	10 (25.6%)	21 (25.0%)	
	T4	1 (2.6%)	16 (19.0%)	
ypN Stage	N0	31 (79.5%)	48 (57.1%)	0.107
	N1	6 (15.4%)	23 (27.4%)	
	N2	1 (2.6%)	5 (6.0%)	
	N3	1 (2.6%)	8 (9.5%)	
ypTNM Stage	Stage 0	5 (12.8%)	2 (2.4%)	**0** **.** **007**
	Stage I	19 (48.7%)	25 (29.8%)	
	Stage II	7 (17.9%)	21 (25.0%)	
	Stage III	8 (20.5%)	36 (42.9%)	
TRG (Ryan)	0	4 (10.3%)	4 (4.8%)	0,199
	1	12 (30.8%)	24 (28.6%)	
	2	17 (43.6%)	29 (34.5%)	
	3	6 (15.4%)	27 (32.1%)	

Variables	Parameters	Mean ± sd	Mean ± sd	*p* ^b^
BMI	Kg/m^2^	26.69 ± 3.05	27.45 ± 3.76	0.275

Variables	Parameters	Median (IQR)	Median (IQR)	*p* ^c^
Age	years	66 (60–73)	63 (62–66)	0.104
Hb	g/dL	12.60 (12.40–13.60)	12.90 (12.60–13.30)	0.699
Alb	g/dL	4.16 (4.00–4.25)	4.19 (4.07–4.40)	0.212
NLR	Ratio	2.41 (1.93–2.66)	2.62 (2.27–3.02)	0.332
PNI	Index	52.10 (49.00–54.30)	53.35 (52.25–55.05)	0.293
Operation Time	Minutes	63 (60–66)	24 (24–25)	**<0** **.** **001**
DFAV	Cm	220 (210–260)	212.50 (205–220)	**0** **.** **041**
RTI	Days	6 (6–7)	6 (6–7)	0.688
LOS	Days	11 (9–14)	8 (8–9)	**0** **.** **007**

TNT, total neoadjuvant therapy; CRT, chemoradiotherapy; ASA, American Society of Anesthesiologists; HT, hypertension; DM, diabetes mellitus; COPD, chronic obstructive pulmonary disease; CAD, coronary artery disease; BMI, body mass index; Hb, hemoglobin; Alb, albumin; NLR, neutrophil-to-lymphocyte ratio; PNI, Prognostic Nutritional Index; RTI, radiotherapy-to-surgery interval; DFAV, distance from anal verge; LOS, length of hospital stay; TRG, tumor regression grade; IQR, interquartile range; SD, standard deviation.

a Chi-Square Test.

b Independent Samples *T-*Test.

c Mann–Whitney *U*-Test.

Bold *p* values indicate statistical significance (*p* < 0.05).

To evaluate overall anastomotic leakage relation with PNI, patients were stratified by using cutoff value of 51.25 and categorized into low-PNI (<PNI) (*n* = 33, 26.8%) and high-PNI (PNI≥**)** (*n* = 90, 73.2%) groups. The proportion of cN1 disease was significantly higher in the <PNI group (51.5% vs. 21.1%; *p* = 0.006). In line with this finding, clinical TNM staging showed a higher rate of Stage III disease in the <PNI group (57.6% vs. 37.8%), whereas Stage II disease was more predominant in the PNI **≥** group (62.2% vs. 42.4) (*p* = 0.049).

With respect to the type of neoadjuvant treatment, the CRT rate was higher in the <PNI group (45.5% vs. 26.7%), while total neoadjuvant therapy was more frequently administered in the PNI **≥** group (73.3% vs. 54.5) (*p* = 0.047). The median age was significantly higher in the PNI **≥** group (64 vs. 62 years, *p* < 0.001). When surgical variables (including transfusion requirement, operative time, and distance from the anal verge), as well as other demographic, clinical, and pathological variables were compared, similar distributions were observed between the groups (*p* > 0.05) (Table 4).

**Table 4:** Comparison of demographic, clinical, and pathological characteristics between low and high prognostic nutritional index groups based on the optimal cutoff value for overall anastomotic leakage (PNI = 51.25).

Variables	Parameters	PNI < 51.25 (*n* = 33)	PNI**≥**51.25 (*n* = 90)	*p* ^a^
		(*n*,%)	(*n*,%)	
Gender	Male	16 (51.6)	57 (62.0)	0.284
	Female	15 (48.4)	35 (38.0)	
ASA Score	ASA 1	4 (12.9)	8 (8.7)	0.328
	ASA 2	10 (32.3)	41 (44.6)	
	ASA 3	15 (48.4)	42 (45.7)	
	ASA 4	2 (6.5)	1 (1.1)	
Smoking	Yes	8 (25.8)	34 (37.0)	0.161
HT	Yes	12 (38.7)	41 (44.6)	0.616
DM	Yes	6 (19.4)	24 (26.1)	0.619
COPD	Yes	2 (6.5)	9 (9.8)	0.726
CAD	Yes	6 (19.4)	18 (19.6)	1.000
Surgery	Laparoscopic	18 (58.1)	60 (65.2)	0.695
	Open	13 (41.9)	32 (34.8)	
Transfusion	Yes	4 (12.9)	9 (9.8)	0.746*
cT Stage	T1	0 (0.0)	1 (1.1)	0.918
	T2	2 (6.5)	8 (8.7)	
	T3	22 (71.0)	66 (71.7)	
	T4	7 (22.6)	17 (18.5)	
cN Stage	N0	14 (45.2)	56 (60.9)	**0** **.** **006**
	N1	15 (48.4)	21 (22.8)	
	N2	0 (0.0)	9 (9.8)	
	N3	2 (6.5)	6 (6.5)	
cTNM Stage	Stage II	14 (45.2)	56 (60.9)	**0** **.** **049**
	Stage III	17 (54.8)	36 (39.1)	
Neoadjuvant	CRT	15 (48.4)	24 (26.1)	**0** **.** **047**
	TNT	16 (51.6)	68 (73.9)	
ypT Stage	T0	1 (3.2)	8 (8.7)	0.408
	T1	8 (25.8)	23 (25.0)	
	T2	11 (35.5)	24 (26.1)	
	T3	6 (19.4)	25 (27.2)	
	T4	5 (16.1)	12 (13.0)	
ypN Stage	N0	21 (67.7)	58 (63.0)	0.726
	N1	8 (25.8)	21 (22.8)	
	N2	1 (3.2)	5 (5.4)	
	N3	1 (3.2)	8 (8.7)	
ypTNM Stage	Stage 0	1 (3.2)	6 (6.5)	0.525
	Stage I	14 (45.2)	30 (32.6)	
	Stage II	6 (19.4)	22 (23.9)	
	Stage III	10 (32.3)	34 (37.0)	
TRG (Ryan)	0	1 (3.2)	7 (7.6)	0.435
	1	12 (38.7)	24 (26.1)	
	2	11 (35.5)	35 (38.0)	
	3	7 (22.6)	26 (28.3)	

Variables	Parameters	Mean ± sd	Mean ± sd	*p* ^b^
BMI	Kg/m^2^	27.52 ± 27.40	27.10 ± 27.05	0.637

Variables	Parameters	Median (IQR)	Median (IQR)	*p* ^c^
Age	years	62 (58–71)	64 (63–69)	**<0** **.** **001**
Hb	g/dL	12.6 (12.0–13.7)	12.8 (12.6–13.3)	0.458
Alb	g/dL	4.18 (3.93–4.36)	4.19 (4.05–4.32)	0.446
NLR	Ratio	2.64 (2.33–3.33)	2.36 (2.00–2.67)	0.788
Operation Time	Minutes	230 (210–260)	210 (210–225)	0.141
DFAV	Cm	6 (6–8)	6 (6–7)	0.151
RTI	Days	31 (26–58)	26 (25–29)	0.910
LOS	Days	11 (10–15)	8 (8–9)	0.071

PNI, Prognostic Nutritional Index; TNT, total neoadjuvant therapy; CRT, chemoradiotherapy; ASA, American Society of Anesthesiologists; HT, hypertension; DM, diabetes mellitus; COPD, chronic obstructive pulmonary disease; CAD, coronary artery disease; BMI, body mass index; Hb, hemoglobin; Alb, albumin; NLR, neutrophil-to-lymphocyte ratio; RTI, radiotherapy-to-surgery interval; DFAV, distance from anal verge; LOS, length of hospital stay; TRG, tumor regression grade; IQR, interquartile range; SD, standard deviation.

a Chi-Square Test.

b Independent Samples *T*-Test.

c Mann–Whitney *U-*Test.

Bold *p* values indicate statistical significance (*p* < 0.05).

All clinically relevant preoperative and perioperative variables were entered into univariate logistic regression analysis to identify potential predictors of anastomotic leakage. In the analysis, distance from anal verge (OR = 0.669, 95% CI: 0.465–0.962, *p* = 0.030), operation time (OR = 1.014, 95% CI: 1.003–1.025, *p* = 0.013), hemoglobin level (OR = 0.688, 95% CI: 0.492–0.961, *p* = 0.028), PNI (OR = 0.845, 95% CI: 0.763–0.936, *p* = 0.001), CAD (OR = 2.985, 95% CI: 1.027–8.679, *p* = 0.045), neoadjuvant therapy type (OR = 0.205, 95% CI: 0.073–0.573, *p* = 0.003), and preoperative blood transfusion (OR = 6.396, 95% CI: 1.861–21.981, *p* = 0.003) were the significant factors associated with anastomotic leakage. Multivariate logistic regression analysis revealed PNI (OR = 0.809, 95% CI: 0.695–0.941, *p* = 0.006), CAD (OR = 4.987, 95% CI: 1.164–21.368, *p* = 0.030), and preoperative blood transfusion (OR = 12.134, 95% CI: 1.634–90.136, *p* = 0.015) as independent risk factors for anastomotic leakage.

These findings indicate that PNI is a significant independent predictor of anastomotic leakage, with each 1-unit increase in PNI conferring a 19.1% reduction in leakage risk. Although conventional chemoradiotherapy demonstrated a protective effect in univariate analysis, this association did not remain significant after adjusting for confounders. Additionally, the presence of CAD increased the risk approximately 5-fold, and preoperative blood transfusion increased the risk 12-fold (Table 5).

**Table 5:** Univariate and multivariate analysis of independent risk factors for AL.

Variables		Univariate Analysis			Multivariate Analysis		
	Parameters	OR	95% CI	*p*	OR	95% CI	*p*
Age	years	1.027	0.979–1.077	0.281	–	–	–
Gender	Male	1.383	0.518–3.695	0.518	–	–	–
BMI	Kg/m^2^	0.945	0.820–1.090	0.440	–	–	–
DFAV	Cm	0.669	0.465–0.962	**0.030**	0.612	0.344–1.090	0.095
Operation Time	Minutes	1.014	1.003–1.025	**0.013**	1.012	0.996–1.029	0.135
Hb	g/dL	0.688	0.492–0.961	**0.028**	0.858	0.548–1.346	0.505
PNI		0.845	0.763–0.936	**0.001**	0.809	0.695–0.941	**0.006**
NLR		0.918	0.650–1.298	0.630	–	–	–
ASA Score				0.168	–	–	–
	ASA2	0.687	0.065–7.253	0.755	–	–	–
	ASA3	2.993	0.344–25.028	0.325	–	–	–
	ASA4	NA	NA	0.999	–	–	–
HT	Yes	1.576	0.591–4.206	0.364	–	–	–
DM	Yes	0.800	0.244–2.627	0.713	–	–	–
COPD	Yes	2.250	0.539–9.389	0.266	–	–	–
CAD	Yes	2.985	1.027–8.679	**0.045**	4.987	1.164–21.368	**0.030**
Smoking	Yes	1.936	0.719–5.215	0.191	–	–	–
Surgery Type	Laparoscopic	0.722	0.129–4.053	0.712	–	–	–
Neoadjuvant Therapy	TNT	0.205	0.073–0.573	**0.003**	0.270	0.068–1.065	0.061
cTNM Stage	Stage II	2.030	0.753–5.470	0.162	–	–	–
PBT	unite	6.396	1.861–21.981	**0.003**	12.134	1.634–90.136	**0.015**

BMI, body mass index; DFAV, distance from anal verge; Hb, hemoglobin; PNI, Pr*o*gnostic Nutritional Index; NLR, neutrophil-to-lymphocyte ratio; ASA, American Society of Anesthesiologists; HT, hypertension; DM, diabetes mellitus; COPD, chronic obstructive pulmonary disease; CAD, coronary artery disease; PBT: Preoperative Blood Transfusion; OR: Odds Ratio, CI: Confidence Interval.

Bold *p* values indicate statistical significance (*p* < 0.05).

## 4 Discussion

This study demonstrated that preoperative PNI is a significant independent predictor of anastomotic leakage in patients with locally advanced rectal cancer who underwent low anterior resection following neoadjuvant therapy. ROC analysis revealed that PNI had a moderate predictive value for anastomotic leakage (AUC = 0.756), with an optimal cutoff value of 51.25 yielding a sensitivity of 71.2% and specificity of 57.9%. In multivariate logistic regression analysis, each 1-unit increase in PNI was associated with a 19.1% reduction in anastomotic leakage risk (OR = 0.809, 95% CI: 0.695–0.941, *p* = 0.006). Additionally, coronary artery disease (OR = 4.987, 95% CI: 1.164–21.368, *p* = 0.030) and preoperative blood transfusion (OR = 12.134, 95% CI: 1.634–90.136, *p* = 0.015) were identified as independent risk factors, increasing the anastomotic leakage risk approximately 5-fold and 12-fold, respectively. Notably, although univariate analysis suggested a protective effect of conventional CRT compared to TNT (OR = 0.205, *p* = 0.003), this association did not remain statistically significant in multivariate analysis (OR = 0.270, *p* = 0.061), indicating that the type of neoadjuvant protocol may not independently influence anastomotic leakage risk when other confounders are accounted for. These findings highlight the critical importance of preoperative nutritional and immunological assessment, asin predicting anastomotic complications in this high-risk patient population undergoing multimodal treatment for rectal cancer. To contextualize these findings, it is important to consider the pathophysiological consequences of neoadjuvant therapy on nutritional and immunological reserves, and the role of these factors in anastomotic healing.

The management of locally advanced rectal cancer has evolved significantly over the past decades, with neoadjuvant therapy becoming an integral component of multimodal treatment strategies. Conventional long-course chemoradiotherapy, consisting of fluoropyrimidine-based chemotherapy with concurrent pelvic radiotherapy, has been the standard of care for achieving tumor downstaging and improving local control. More recently, total neoadjuvant therapy has emerged as an alternative approach, incorporating induction or consolidation chemotherapy in addition to chemoradiotherapy, with the aim of enhancing systemic disease control and improving pathological complete response rates. However, these intensive treatment regimens are not without consequences on the patient's physiological reserve. Chemoradiotherapy is known to induce significant immunosuppression, primarily through lymphocyte depletion, which may persist for weeks to months following treatment completion (5). Furthermore, treatment-related toxicities such as mucositis, nausea, anorexia, and diarrhea can lead to inadequate nutritional intake and subsequent malnutrition. The prolonged treatment duration associated with TNT protocols may exacerbate these effects, resulting in cumulative deterioration of both immunological and nutritional status (22). Given that adequate tissue perfusion, immune competence, and nutritional reserves are essential prerequisites for optimal anastomotic healing, the assessment of these parameters prior to surgery becomes critically important in patients who have undergone neoadjuvant therapy (23).

In this context, the Prognostic Nutritional Index emerges as a particularly relevant biomarker for patients undergoing neoadjuvant therapy. PNI, calculated from serum albumin and peripheral lymphocyte count, simultaneously captures both the nutritional and immunological dimensions that are adversely affected by chemoradiotherapy. Serum albumin, as the primary component of PNI, reflects hepatic synthetic function, overall nutritional status, and serves as a surrogate marker for protein reserves essential for tissue repair and collagen synthesis at the anastomotic site (24). The lymphocyte count, the second component of PNI, represents the cellular arm of adaptive immunity, which plays a crucial role in infection control, inflammatory regulation, and wound healing processes (25). Therefore, a low PNI value in patients who have completed neoadjuvant therapy may indicate a compromised physiological state characterized by depleted protein reserves and impaired immune function, both of which are fundamental prerequisites for successful anastomotic healing. Furthermore, the simplicity and accessibility of PNI calculation from routine preoperative blood tests make it an attractive tool for clinical risk stratification, allowing surgeons to identify high-risk patients who may benefit from preoperative nutritional optimization or closer postoperative surveillance (24, 26, 27).

Our findings are consistent with previous studies demonstrating the predictive value of PNI for anastomotic leakage in colorectal surgery. Moro-Valdezate et al. reported that PNI was an independent predictor of anastomotic leakage following curative colorectal cancer surgery (RR: 0.151, 95% CI: 0.036–0.640). Similarly, Zhang et al. found that PNI had moderate predictive value for anastomotic leakage in colorectal cancer patients (AUC = 0.625). However, these studies included heterogeneous populations comprising both colon and rectal cancer patients with varying treatment protocols. Our study specifically focused on rectal cancer patients who underwent standardized neoadjuvant therapy, providing more homogeneous data for this high-risk subgroup. The optimal PNI cutoff value of 51.25 identified in our study is comparable to previously reported thresholds ranging from 45 to 52 in colorectal cancer literature.

Several studies have investigated the relationship between PNI and survival outcomes in rectal cancer patients. Tokunaga et al. demonstrated that patients with PNI values below 46.94 had significantly worse survival outcomes; however, their study did not specifically evaluate anastomotic leakage as an endpoint. Nevertheless, they reported that Clavien-Dindo grade ≥2 complications occurred more frequently in patients below their proposed PNI cutoff. Notably, not all patients in their cohort received neoadjuvant therapy. In a separate study by Miyata et al. examining five different prognostic markers in 131 patients, no significant association was found between PNI and anastomotic leakage. However, approximately one-fifth of their patients did not receive neoadjuvant therapy, and patients who underwent abdominoperineal resection were also included in the analysis. Brisinda et al., in a large cohort study using the commonly referenced cutoff value of 40, demonstrated that anastomotic leakage occurred significantly more frequently in patients with PNI <40 compared to those with higher values (18.2% vs. 6.6%; *p* < 0.001). However, neoadjuvant therapy was not administered to all patients in their cohort either. These methodological differences—including heterogeneous neoadjuvant treatment protocols and inclusion of non-restorative procedures—may partially explain the inconsistent findings across studies and highlight the strength of our homogeneous study population receiving standardized neoadjuvant therapy (28–30).

From a clinical perspective, PNI offers a practical and easily accessible tool for preoperative risk stratification in rectal cancer patients undergoing neoadjuvant therapy. Calculated using only serum albumin and peripheral lymphocyte count—parameters routinely obtained in standard preoperative evaluation—PNI requires no additional cost or invasive procedures. Patients identified with low PNI values prior to surgery may benefit from targeted nutritional optimization, including dietary counseling, oral nutritional supplements, or in select cases, immunonutrition formulas. Furthermore, in patients with suboptimal PNI, surgeons may consider protective diverting stoma more liberally or allow additional time for nutritional rehabilitation before proceeding with restorative surgery. Integrating PNI into preoperative assessment protocols could facilitate individualized treatment planning and potentially reduce anastomotic leakage rates in this high-risk population (31–34).

### 4.1 Limitations and strengths

This study has several limitations. The retrospective design inherently carries risks of selection bias and unmeasured confounding variables. Additionally, the relatively limited sample size may have reduced statistical power to detect associations with less common variables. However, these limitations stem partly from the strict inclusion criteria necessary to ensure methodological rigor. Including only patients who received neoadjuvant therapy, applying standardized surgical techniques, and establishing homogeneous patient selection criteria were essential to minimize confounding factors and strengthen the validity of our findings. Furthermore, anastomotic leakage is a relatively rare complication, making large sample sizes challenging to achieve in single-center studies with stringent inclusion criteria. Notably, to our knowledge, this is the first study to evaluate PNI as a predictor of anastomotic leakage exclusively in rectal cancer patients who have undergone neoadjuvant therapy—a population with distinct nutritional and immunological vulnerabilities. Additionally, PNI was assessed at a single preoperative timepoint in this study; future research incorporating dynamic evaluation of nutritional and immunological parameters across the neoadjuvant treatment period may further enhance the predictive accuracy of anastomotic leakage risk stratification. Future multicenter prospective studies with larger cohorts are warranted to validate these findings and establish standardized PNI cutoff values for clinical application.

## 5 Conclusions

Preoperative PNI is an independent predictor of anastomotic leakage in locally advanced rectal cancer patients treated with neoadjuvant therapy. With an optimal cutoff value of 51.25, PNI provides a simple, cost-effective, and readily available tool for preoperative risk stratification. Incorporating PNI into routine preoperative assessment may help identify high-risk patients who could benefit from nutritional optimization or more liberal use of protective diverting stoma. Future prospective multicenter studies are needed to validate these findings.

## Data Availability

The raw data supporting the conclusions of this article will be made available by the authors, without undue reservation.
